# Leveraging the potential of machine learning for assessing vascular ageing: state-of-the-art and future research

**DOI:** 10.1093/ehjdh/ztab089

**Published:** 2021-10-18

**Authors:** Vasiliki Bikia, Terence Fong, Rachel E Climie, Rosa-Maria Bruno, Bernhard Hametner, Christopher Mayer, Dimitrios Terentes-Printzios, Peter H Charlton

**Affiliations:** 1 Laboratory of Hemodynamics and Cardiovascular Technology (LHTC), Swiss Federal Institute of Technology, CH-1015 Lausanne, Vaud, Switzerland; 2 Baker Heart and Diabetes Institute, 75 Commercial Rd, Melbourne, Victoria, 3004 Australia; 3 Department of Cardiometabolic Health, Melbourne Medical School, University of Melbourne, Grattan Street, Parkville, Victoria, 3010 Australia; 4 Université de Paris, INSERM U970, Paris Cardiovascular Research Centre, Integrative Epidemiology of Cardiovascular Disease, Paris, France; 5 Center for Health & Bioresources, AIT Austrian Institute of Technology, Giefinggasse 4, 1210 Vienna, Austria; 6 First Department of Cardiology, Hippokration Hospital, Medical School, National and Kapodistrian University of Athens, 114 Vasilissis Sofias Avenue, 11527, Athens, Greece; 7 Department of Public Health and Primary Care, Strangeways Research Laboratory, 2 Worts' Causeway, Cambridge, CB1 8RN, UK; 8 Research Centre for Biomedical Engineering, City, University of London, Northampton Square, London, EC1V 0HB, UK

**Keywords:** Arterial stiffness, Blood pressure, Cardiovascular, Central blood pressure, Pulse wave velocity, Machine learning

## Abstract

Vascular ageing biomarkers have been found to be predictive of cardiovascular risk independently of classical risk factors, yet are not widely used in clinical practice. In this review, we present two basic approaches for using machine learning (ML) to assess vascular age: parameter estimation and risk classification. We then summarize their role in developing new techniques to assess vascular ageing quickly and accurately. We discuss the methods used to validate ML-based markers, the evidence for their clinical utility, and key directions for future research. The review is complemented by case studies of the use of ML in vascular age assessment which can be replicated using freely available data and code.

## Graphical Abstract

**Figure:**
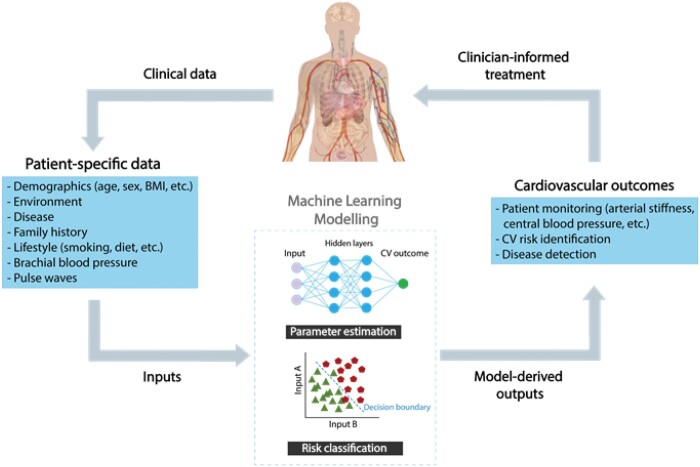

## Introduction

Age is a key risk factor for hypertension and cardiovascular disease (CVD).^1^ A major consequence of ageing is the progressive stiffening of the major arteries, particularly the proximal aorta. In an optimally functioning cardiovascular system, the elastic properties of the large arteries ensure that the pulsatile pressure and flow generated by left ventricular ejection is dampened, minimizing potential harm to the microvasculature. However, the cushioning (elastic) properties of the large arteries diminish with age giving rise to arterial stiffening. While age-related arterial damage occurs predominantly in later life, there is wide variability between individuals, with some displaying early vascular ageing.^2^ This has led to the concept that vascular age, as opposed to chronological age, may be better related to the prognosis of CVD.^3^

Arterial stiffness is a promising marker of vascular ageing and many studies have shown that the stiffness of the large arteries is related to elevated CVD risk in adults, independently of traditional cardiovascular risk factors.^4^ Given the world’s ageing population, effective monitoring of vascular ageing is increasingly important, and clinical biomarkers that can accurately describe the status of the vasculature are highly desirable.^5^ A commonly used index of arterial stiffness is carotid-femoral pulse wave velocity (cfPWV), the speed at which the pressure wave travels through the arteries, typically measured via applanation tonometry.^6^ Central (aortic) blood pressure (CBP), the pressure the heart, and central organs are exposed to, is also indicative of vascular ageing and is related to cardiovascular events and mortality^7^^,^^8^ independently of brachial blood pressure (BP).^9^ Several other indices can also be used to assess vascular age including cellular biomarkers, coronary artery calcium scores, endothelium function, carotid intima-media thickness, and atherosclerosis indices. This review focuses on arterial stiffness biomarkers such as pulse wave velocity (PWV), given the wealth of evidence that they can capture age-related arteriosclerotic changes.

**Figure 1:**
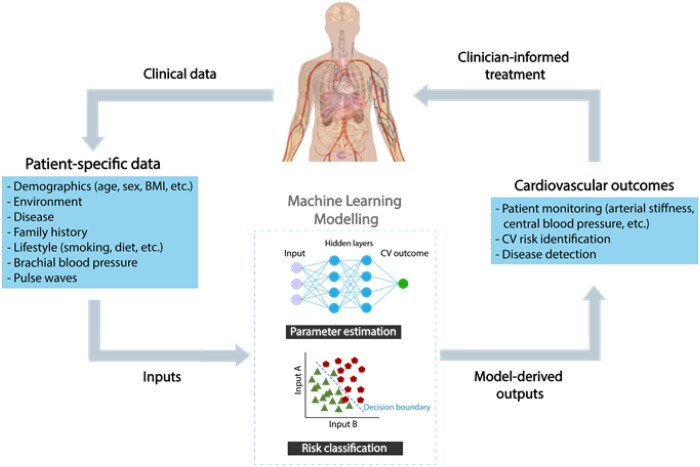
Using machine learning to assess vascular ageing biomarkers from more easily obtained measurements. BMI, body mass index; CV, cardiovascular; 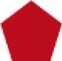, presence of CV event; 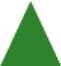, absence of CV event. Adapted from: ‘Adult male with organs’, Wikimedia Commons, under CC0 1.0.

Machine learning (ML) provides systems or models with the capacity to learn automatically from data without explicit human input. Recent technological advances have spurred an abundance of ‘big data’ in healthcare:^10^ data of ‘such a high volume, velocity (i.e. rate of collection), and variety (i.e. different types of variables) to require specific technology and analytical methods for its transformation into value’.^11^ Machine learning algorithms, including deep learning algorithms (a subset of ML), are being used increasingly due to their flexible nature in evaluating large datasets without the need for specified assumptions. Since the distinction between ML and statistical modelling is not clear-cut,^12^ this review incorporates both ML and statistical modelling techniques. ML is now being used to develop new methods for assessing vascular age which may be more accurate or simpler than existing methods. For example, multiple linear regression has been used to develop a model to estimate PWV from age and routine BP measurements, and the result is predictive of outcomes.^13^^,^^14^ Machine learning has also been used to develop models to estimate CBP from peripheral pressure waves, including using a generalized transfer function to estimate a central pressure wave from a peripheral wave,^15^ and using regression analysis to estimate CBP from brachial BP and PWV.^16^ This critical review highlights relevant ML techniques, their clinical utility, and directions for future research to leverage the potential of ML for assessing vascular ageing (*Figure  1*).

## The role of machine learning in assessing vascular age

### Using machine learning to assess vascular age

Machine learning has been used to develop two types of models to assess vascular age: parameter estimation models and risk classification models. Parameter estimation models estimate a target parameter from more easily obtained measurements, such as estimating PWV from age and BP. Risk classification models classify a subject according to their risk of a particular outcome or diagnosis, such as being at high or low risk of cardiovascular (CV) events. *Table 1* provides examples of clinical applications of these two types of models, detailing the ML techniques used in each case.

**Table 1:** Applications of statistical modelling and machine learning in vascular age assessment

Type of model	ML techniques	Applications
Parameter estimation	Simple linear regression	Estimating carotid AI from radial AI^17^ (mean error: −4 ± 23%, *R*^2^ = 0.66)
	Transfer function	Estimate CBP from a cuff BP and peripheral pressure pulse waves^15^ [mean error: 4.49 (−6.06, −2.92) mmHg]
	Multiple linear regression	Estimating PWV from age and BP (developed in,^18^ and applied in^13^) [mean error: −0.3% (−15%, +17%)]
		Estimating age from non-invasive CV parameters^19^ (men: MAE = 6.91 years, *R*^2^ = 0.55, women: MAE = 5.87 years, *R*^2^ = 0.69)
	Gaussian process regression	Estimating PWV and BP from PTT and features derived from non-invasive pulse waves^20^ (PWV: *R*^2^ = 0.88, SBP: *R*^2^ = 0.56, DBP: *R*^2^ = 0.87)
	Neural network	Estimating systolic CBP from radial systolic and diastolic BPs^21^ (*R^2^* = 0.94, mean error: −0.1 ± 3.9 mmHg)
		Estimating ankle-brachial index from a PPG pulse wave^22^ (precision/sensitivity: 97.7%/97.1%)
		Estimating PWV and BP from either PPG pulse waves, or features derived from PPG pulse waves^23^ (PWV: *R*^2^ = 0.93, SBP: *R*^2^ = 0.80, DBP: *R*^2^ = 0.92)
	Ensemble of neural networks	Estimating age from blood test results^24^ (*R^2^* = 0.82, MAE = 5.55 years)
		Estimating PWV from routine clinical variables and an uncalibrated carotid tonometry waveform^25^ (mean error: 0.00 ± 2.07 m/s, *r* = 0.72)
Risk classification	Decision tree	Predicting who will suffer a CV event by combining routinely measured and blood test data, and non-invasive CV parameters^26^ (sensitivity/specificity: 98%/95%)
		Classifying subjects as high or low risk for CV events using risk factors and parameters derived from carotid ultrasound images^27^ (sensitivity/specificity: 9.5%/96.5% and 5.5%/99%)
		Predicting the presence of obstructive coronary artery disease from clinical data and the coronary artery calcium score^28^ (sensitivity/specificity: 78%/62.8% and 80%/81.5%)
		Predicting the presence of coronary heart disease from PWV and clinical and laboratory parameters^29^ (sensitivity/specificity: 82%/85%)
	Support vector machine	Predicting who will suffer a CV event from risk factors^30^ (sensitivity/specificity: 86%/95%)
		Classifying a set of pulse wave features as ‘young’ or ‘old’,^31^ or ‘high’ or ‘low’ PWV^32^ (sensitivity/specificity: 93%/78%)
	Neural network	Predicting coronary heart disease from clinical data, haemodynamic data, and PWV^33^ (sensitivity/specificity: 80%/92%)
	Ensemble of ML pipelines	Predicting CV events from biobank variables (including many which are not routinely recorded)^34^ (sensitivity/specificity: 69.9%/—)

AI, augmentation index; BP, blood pressure; CBP, central blood pressure; CV, cardiovascular; DBP, diastolic BP; MAE, mean absolute error; ML, machine learning; PPG, photoplethysmogram; PTT, pulse transit time; PWV, pulse wave velocity; *R*^2^, coefficient of determination; SBP, systolic blood pressure.

The ML techniques used in vascular age assessment are predominantly ‘supervised’ techniques—i.e. they learn how to generate an output (a parameter or risk class) by learning from training input data which are labelled with reference outputs. For instance, a model for estimating PWV from age and BP can be developed using training data consisting of the required inputs (age and BP) and desired outputs (PWV values).^18^  *Table 2* provides details of the capabilities of supervised ML techniques, allowing one to choose an appropriate technique for a particular application. The choice of ML technique is determined by the type of output required (a parameter or a risk class) and the nature of the input data (single, multiple, or waveform inputs). Often more than one technique is suitable for a particular problem, in which case the choice can be informed by the pros and cons of using each technique.^35^

**Table 2:** The Capabilities of selected statistical modelling and supervised machine learning techniques

ML technique	Capabilities				
	Output type		Input type		
	Parameter estimation	Risk classification	Single input	Multiple inputs	Waveform input
Simple linear regression	✓^17^	X	✓^17^	X	X
Transfer function	✓^15^	X	✓^15^	X	✓^15^
Multiple linear regression	✓^18^	X	X	✓^18^	X
Gaussian process regression	✓^20^	X	X	✓^20^	X
Neural network	✓^21^	✓^33^	X	✓^21^	✓^22^
Decision tree	✓	✓^26^	X	✓^26^	X
Support vector machine	X	✓^30^	X	✓^30^	X

Model types: (i) parameter estimation—estimating a vascular ageing parameter (such as central blood pressure) from more easily obtained measurements; (ii) risk classification—categorizing patients according to whether or not they are likely to experience an event, or the presence or absence of a diagnosis.

Input types: (i) single input—a single numerical value (e.g. age); (ii) multiple inputs; (iii) waveform input—whether or not the ML technique can accept a waveform as one of the inputs (e.g. a pulse wave).

ML, machine learning.

### Opportunities

Machine learning provides opportunities to enhance vascular age assessment through the analysis of complex datasets, digital signals, and images. In research, ML is now widely used, aided by large datasets and high-performance computing systems. In clinical practice, ML-based technologies present opportunities to improve the accessibility and performance of vascular age assessments. These opportunities are now discussed.

#### Data availability

A large amount of biomedical and clinical data is routinely collected which is suitable for training ML models to assess vascular age. Advances in measurement techniques and systems have allowed for the acquisition of high-fidelity data suitable for assessing vascular age. Arterial pulse wave signals can be acquired in specialist clinics using, for instance, applanation tonometry and ultrasound. Additional signals such as the electrocardiogram (ECG), ballistocardiogram, and photoplethysmogram (PPG) can be acquired by consumer devices such as smartphones and fitness trackers. Images of the cardiovascular system and affected organs can be acquired by ultrasound, magnetic resonance imaging, and computed tomography, resulting in improved visual assessment of functional and structural changes associated with disease and pathology. The multifaceted nature and high dimensionality of such data is the primary driving force in cardiovascular Big Data.^36^ Additionally, the complexity of the data often renders traditional statistical methods insufficient to efficiently develop predictive tools to assist clinical decision-making. In contrast, ML offers promise for developing methods to improve and automate cardiovascular health assessment, and to guide therapeutic interventions.

#### Computing systems

Recent years have seen rapid advancements in both hardware and software.^37^ The refinement of hardware components, such as high-performance processors and graphics processing units, has reduced the computational time required to train an ML model, even with large datasets. Additionally, many ML techniques are widely available in software packages such as Python and MATLAB. These advances make it practical for researchers to use ML routinely.

#### Improving the accessibility of vascular age assessment

Machine learning-based techniques for assessing vascular age have potential to improve the accessibility of vascular age assessment. Currently, BP is the only biomarker of vascular age which is routinely measured in primary care. A number of issues limit the use of other markers of vascular ageing.^5^ While cfPWV has satisfactory repeatability,^38^ its measurement requires a skilled operator, and alternative PWV measurements which can be obtained more easily may not reflect the status of the aorta as precisely, such as carotid-radial PWV^39^ and PWV assessed from the ECG and a pulse wave.^40^ There is a similar tension between precision and ease of measurement for CBP.^15^^,^^41^ Machine learning-based techniques are now being developed which could be used in primary care with minimal additional workload, such as using routinely collected clinical data to estimate CBP or PWV or assessing vascular age from pulse waves acquired by pulse oximeters (as detailed in the Case Studies below). Thus, ML-based techniques have potential to improve the accessibility of vascular age assessment.

#### Improving the performance of vascular age assessment

Machine learning-based techniques may have potential to provide improved performance over traditional statistical modelling techniques, although this potential has not yet been widely realised.^12^ Machine learning-based methods are particularly well-suited to handling high-volume data including images, time-series, or multi-dimensional data. In such cases, ML can have an immense advantage and offer possibilities far beyond traditional techniques. Some studies have compared the performance of novel ML-based techniques with traditional techniques. For instance, Xiao *et al.*^21^ compared using a neural network to estimate CBP from peripheral pulse waves with the widely used transfer function approach. They did not find a substantial difference in performance between the two approaches. More broadly, ML has been found not to confer benefit over logistic regression for clinical prediction models.^12^ Therefore, despite the current hype around artificial intelligence, there is still uncertainty in whether ML-based methods have an advantage over traditional statistical methods in vascular age assessment. Several ML methods have demonstrated minimal benefit over traditional approaches. In particular, a recent study reported an improvement in the identification of young, asymptomatic individuals with an increased risk of subclinical atherosclerosis.^42^ Another study showed that ML methods offered only limited improvement over traditional logistic regression^43^ (see section Risk classification). In the future, it is likely that ML-based techniques would either have to provide improved performance or facilitate easier measurement, in order to replace traditional statistical approaches.

### Challenges

In this section, we discuss key challenges in developing ML-based techniques for assessing vascular age.

#### Data acquisition

Large datasets are required to develop ML-based techniques. Devices for acquiring arterial pulse waves in the clinic, such as ultrasound and applanation tonometry devices, often output the data in a format suitable for analysis, although they require a skilled operator. On the other hand, consumer devices that measure pulse wave signals (such as smartphones, smartwatches, and fitness trackers) can be used by patients with no need for a skilled operator, but do not routinely record the data for analysis. Those devices which do record pulse waves in everyday life can require much user interaction for reliable data acquisition.^44^ While studies examining PWV exist, these are often limited by small sample size, homogeneity, lack of follow-up with CV events and diverse health profiles. Nonetheless, suitable datasets have previously been acquired in large-scale local and international studies.^18^^,^^45^

The use of reliable datasets is critical for developing accurate and clinically relevant ML models. The following should be considered. First, measurement protocols should be coherent and properly standardized, as the data collection methodology impacts the learning process performed by the models. Second, the measured input and reference data should be of high quality, particularly clinical measurements (such as PPG waveforms, see section Using consumer devices to assess vascular age in daily life) which can be subject to errors due to improper calibration, noise, interference, or artefact. Importantly, the use of unsuitable data can lead to inaccurate outcomes and enable false medical decisions (e.g. in applications of subject classification using risk scores and clinical diagnoses). Additional considerations on the reference techniques can be found in section Validation types of machine learning-based methods.

#### Experimental methodology

A recent review highlighted shortcomings in the methodology used to develop clinical prediction models using ML.^12^ First, few studies used external validation, and many either did not report validation procedures clearly or had potential biases in validation procedures, such as selecting variables on all data or not repeating all modelling steps in the validation. Second, studies commonly assessed performance using the area under the receiver operator curve (AUROC) statistic, but usually did not assess the accuracy of risk estimates.^12^ This recent review provides important guidance, which can inform future studies using ML in vascular ageing assessment.

In addition, the lack of interpretability of ML models has often been considered as a limitation for the use of ML in clinical applications. Although ongoing innovations include establishing new concepts, such as explainable ML^46^ or parallel models, where one is used for core computation and the other for interpretation,^47^^,^^48^ the relevant research is still ongoing. Nonetheless, if ML models are highly accurate and guidelines for the proper clinical use of ML are established, then we might consider using them for specific tasks. Simulated data, generated from a computer model (e.g. the data in the Case Studies below) could aid interpretability, as they are derived from deterministic models in which relationships between variables may be more easily explained.

Care is required to ensure ML models are developed and used appropriately. When developing an ML model, there is a danger of overfitting to the training data, reducing generalizability. Techniques such as feature selection can be used to determine which clinical biomarkers should be included in the model. When using a model, if the input data is of poor quality then the output will be affected, potentially leading to misdiagnosis.^49^ High quality data, which has been captured with clinical aptitude and pre-processed appropriately (i.e. missing values adjusted, data transformation), may lead to better sensitivity and specificity. Hence, guidelines for the use of ML in clinical prediction are warranted, as well as the need to consult with biostatisticians to minimize preventable errors.

#### Reporting machine learning models

The TRIPOD (Transparent Reporting of a multivariable prediction model for Individual Prognosis Or Diagnosis) statement provides a checklist of 22 methodological aspects that should be reported in studies of prediction models.^50^ A new statement specific to ML studies is now being developed.^51^ Even with clear and concise reporting of the methods used to design and validate models, further quality assurance through external validation is required. However, well-grounded external validation studies are sparse as often there is a lack of available data other than that used for model development.^52^ Even with access to sufficiently large datasets, external validation studies are often poorly reported.^53^ It is important that rigorous procedural steps are adhered to during the design, validation, and external validation of ML-based techniques to enhance vascular ageing assessment.

#### Benchmark datasets

Benchmark datasets could provide a standardized approach to developing ML-based techniques for assessing vascular age. Benchmark datasets are datasets that have been chosen to be the ‘standard’ for a model to be evaluated against.^54^ Benchmark datasets should contain data reflective of the target population and ideally contain a wide range of characteristics to allow the strengths and weaknesses of ML-based techniques to be assessed.^55^ To the best of our knowledge, there is no currently known registry or biobank containing a ‘gold standard’ benchmark dataset that may be used for ML studies in vascular age assessment. Hence, future endeavours should consider the establishment of a registry or consortium, containing data with relevant markers of arterial stiffness, that has both adequate sample size and is reflective of the target population.^5^

### Case studies

Case studies of the use of ML in vascular age assessment are now presented. To aid reproducibility, the case studies use publicly available, simulated haemodynamic data for 3837 healthy adult subjects aged from 25 to 75 years old from the Pulse Wave Database.^56^ The simulated subjects all had different cardiovascular properties within normal ranges, including arterial stiffness, BP, aortic diameter, stroke volume, and heart rate (HR). The case studies are each accompanied by a tutorial allowing them to be replicated using the openly available data and source code (as detailed in the Supplementary material online). A case study is now presented on using a random forest regressor to estimate CBP from age, cuff BP, and HR. Two further case studies are provided in the Supplementary material online on: (i) using multiple linear regression to estimate PWV from age and BP and (ii) using a neural network to assess vascular age from pulse waves.

**Figure 2:**
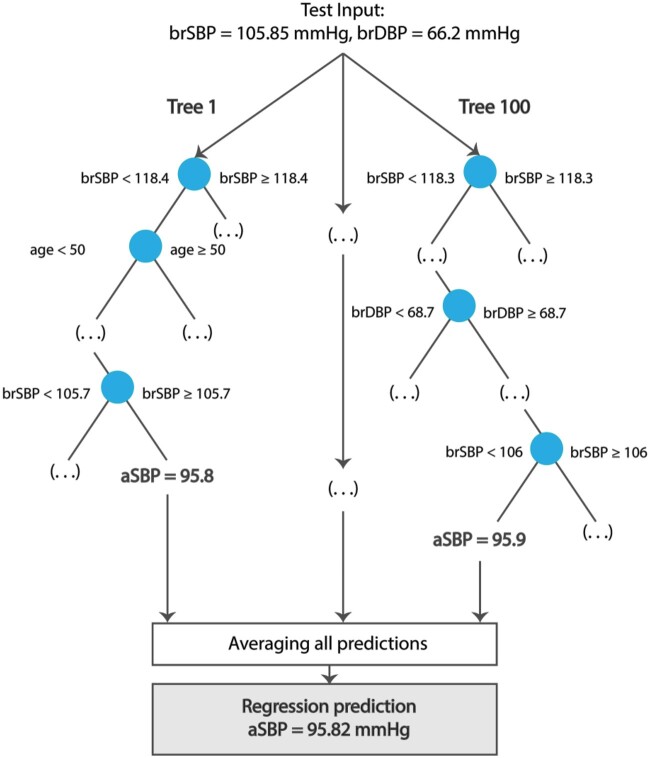
Schematic representation of a random forest regression prediction.

In this case study, central systolic (CSBP) and diastolic BP (CDBP) are estimated from age, brachial (cuff) SBP (BSBP) and DBP (BDBP), and HR using a random forest regressor.^57^ A random forest regressor is an ensemble learning method which consists of a collection of randomized base regression trees. Each tree is built by splitting the source set (the root node of the tree) into branches based on a certain feature of the input variables. This process is repeated recursively until the subset at a node has the same values of the target output variable. The final prediction is provided by averaging the predictions of all the regression trees. The formal structure of a random forest predictor is shown in *Figure  2*. This case study employs two random forest regression models to predict, respectively, CSBP and CDBP (target outputs) from age, BSBP, BDBP, and HR (inputs). The regression models were trained using 60% of the entire population while 20% was kept for testing. Given the importance of an external validation in the design of an ML study, a ‘validation step’ is incorporated in the case study. In particular, we hold out the remaining 20% of the data (referred to as the validation set) and evaluate the performance of the ML regression model on these data. The number of trees of each random forest regressor was set to 100.

**Figure 3:**
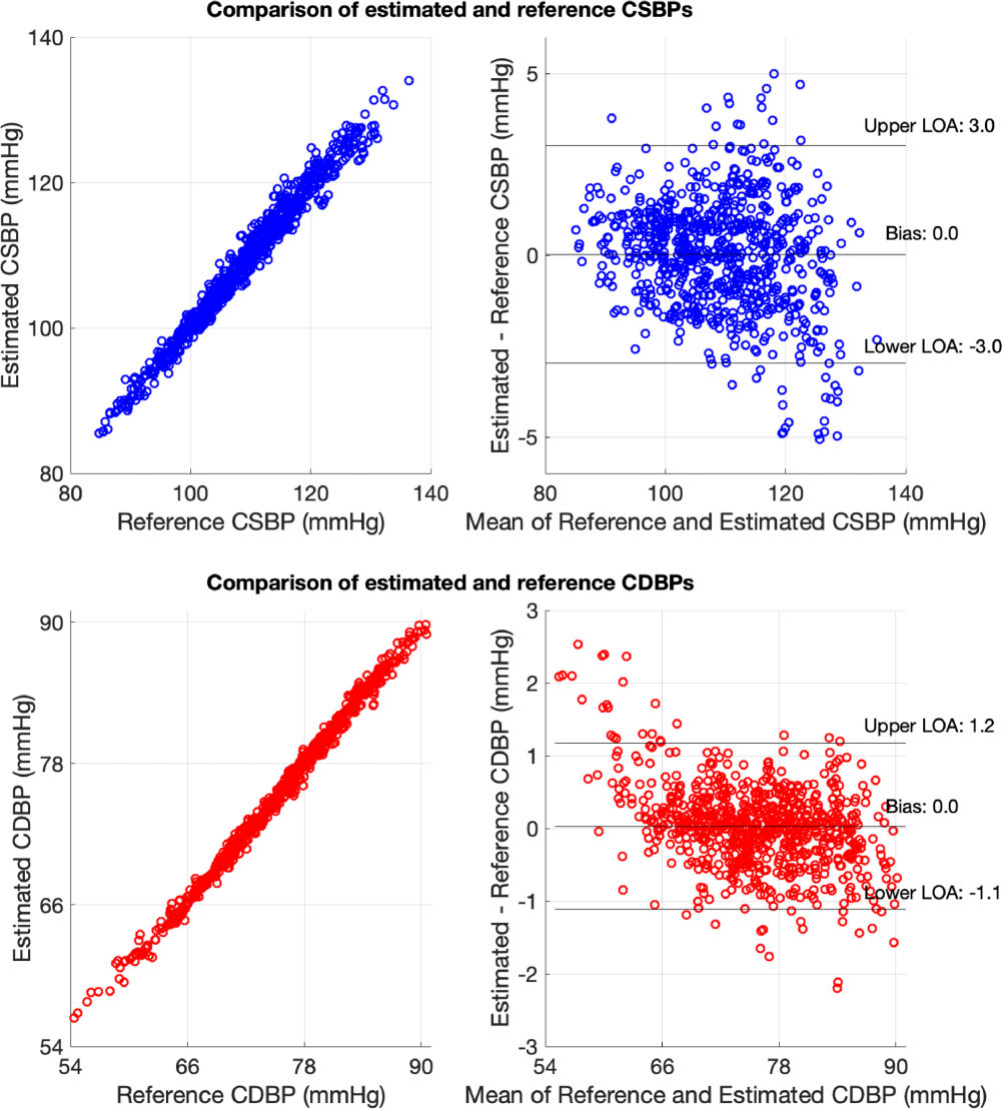
A case study of estimating central systolic blood pressure and central diastolic blood pressure from age, brachial systolic and diastolic blood pressures, and heart rate using a random forest regressor. CDBP, central diastolic blood pressure; CSBP, central systolic blood pressure; LOA, limit of agreement.

The comparison between the estimated CSBP and the reference CSBP is presented in *Figure  3* (top panel). The limits of agreement between the estimated and reference CDBP (this statistic is described in section Estimation of vascular parameters) were narrow at ±3 mmHg. Good performance was also achieved for the estimation of CDBP (see lower panel of *Figure  3*), with limits of agreement of ±1 mmHg.

A similar performance was reported when the models were validated using the validation set. The limits of agreement between the estimated and reference data were found to be equal to ±3 mmHg for CSBP and ±1 mmHg for CDBP, respectively. It should be noted that the simulated data do not permit an actual external validation; yet, this case study aims to demonstrate the practical steps for the proper design of an ML method.

This example demonstrates how ML can potentially be used to transform routine measurements into an additional parameter which is difficult to acquire in practice. This case study indicated that CSBP and CDBP could be estimated precisely from age, brachial BPs, and HR using a random forest regressor. This illustrates a possible application for an ML-based tool in clinical practice.

## Validation of machine learning-based methods

This section presents different types of validation techniques which are commonly used to evaluate the accuracy of an ML model. Subsequently, it summarizes the reported performance of previously developed methods on the estimation of vascular parameters and risk classification.

### Validation types of machine learning-based methods

In ML model studies, the performance of the model is usually assessed using either cross-validation or external validation methods. In cross-validation, the ML model is trained against a subset of the data before being evaluated against the remaining data, and this process is repeated using different subsets of the data.^58^ This technique helps overcome issues such as selection bias or overfitting. However, the model performance needs to be tested for heterogeneity, which is performed through external validation. The use of independent datasets allows proper assessment of whether a model can be generalized to populations outside of the study data.^52^

Many studies, unfortunately, overlook the need to externally validate ML models and often find their reported model performances to be limited to the study-specific population, leading to potentially wasted resources.^59^ However, a recent ML-based study automating phase-contrast cardiovascular magnetic resonance (CMR) aortic flow quantification is one of very few studies to have performed both cross-validation and external validation.^60^ They showed that in-house ML segmentation, using a neural network approach on 190 coronary artery disease patients, was robust, did not require human intervention, and strongly correlated with the manual quantification of an expert CMR reader (*r* > 0.99). When externally validated against two institutionally independent datasets (*n* = 20), ML model performance strongly correlated with manual segmentation (*r* > 0.99). Though their external validation sample size is relatively low, they have reported clear methodology and their findings have potential to be independently tested by other researchers.

Furthermore, attention should be paid to the selection of the technique to be used to acquire the data for the ML modelling. The use of more reliable and thoroughly validated commercial devices should result in a more robust prediction model when compared to a prediction model trained using data from a less validated apparatus. For instance, one such study used an artificial neural network to predict CBP from radial BP measurements.^21^ Central blood pressure estimates may agree more closely with the gold standard of invasive BP, although estimates of brachial cuff BP may be more useful as current clinical guidelines are based on cuff BP data. Similarly, in the case of PWV, reference values have been obtained for cfPWV, and, in this view, ML prediction of cfPWV might be more valuable than prediction of invasive PWV. Hence, one should always consider the current state-of-knowledge and the particular needs of each application and select with caution the data and the design of their ML estimator.

### Estimation of vascular parameters

*Table 3* summarizes the findings of only a limited number of validation studies for the ML estimation of PWV and CBP. Those studies are based on the use of easily obtained clinical data which are transformed into more relevant parameters of vascular ageing. Generally, *in* *vivo* validations demonstrated a good performance in most of the proposed ML methods (*Table 3*). In these studies, the performance of ML-based methods was often assessed using the correlation between estimated and reference parameter values. The limits of agreement technique, also known as Bland–Altman analysis, was also used, although less frequently. This technique quantifies the accuracy and precision of measurements using the bias (mean error) and limits of agreement, which is twice the standard deviation of the errors.^61^ The limits of agreement technique is preferred for assessing agreement between two measurement methods since correlation coefficients can be misleading in this context.^61^

**Table 3:** List of selected validation studies of machine learning techniques compared to reference methods for vascular parameter estimation

Publication	Target parameter	Inputs	Machine learning technique	Sample size	Age (years)	*R* ^2^	Mean error	Externally validated (yes/no)
Greve *et al.* (2016)^13^	cfPWV (Complior)	Age, brachial BP (Cuff)	Multiple linear regression	1045	56 ± 13 (CV event), 50 ± 12 (no CV event)	—	−0.3% (−15%, +17%)	Yes
Huttunen *et al.* (2019)^20^	aPWV^a^	PPG wave^a^	Gaussian process regression	943	—	0.88	—	No
Huttunen *et al.* (2020)^23^	aPWV^a^	PPG wave^a^	Neural network	943	—	0.93	*—*	No
Tavallali *et al*. (2018)^25^	cfPWV (Tonometry)	Carotid BP wave (Tonometry)	Ensemble of neural networks	5020	45 ± 11	0.72	0.00 ± 2.07 m/s	No
Bikia *et al.* (2020)^16^	CSBP (SphygmoCor)	Brachial BP (Cuff), cfPWV (Tonometry)	Supports vector regressor	783	61 ± 11	0.94	0.43 mmHg (−7.88 mmHg, 8.73 mmHg)	No
Huttunen *et al.* (2019)^20^	CSBP, CDBP^a^	PPG wave^a^	Gaussian process regression	943	—	0.56, 0.87	—	No
Huttunen *et al.* (2020)^23^	CSBP, CDBP^a^	PPG wave^a^	Neural network	943	—	0.80, 0.92	—	No
Xiao *et al.* (2017)^21^	CSBP (Invasive)	Radial BP (Invasive)	Neural network	62	61 ± 11	0.94	−0.1 ± 3.9 mmHg	No

aPWV, aortic pulse wave velocity; BP, blood pressure; CDBP, case diastolic BP; cfPWV, carotid-femoral pulse wave velocity; CI, confidence interval; CSBP, central systolic blood pressure; PPG, photoplethysmogram; *R*^2^, coefficient of determination; SD, standard deviation.

a The study population used for the training/testing scheme was generated from a computer simulator. Local aPWV was calculated analytically using the Bramwell–Hill formula.^19^

Although there are not many meta-analyses to systematically compare the performance of ML models with traditional statistical methods for the estimation of vascular parameters, some studies have compared the two approaches. In some cases,^25^^,^^62^ ML models appeared to outperform the traditional prediction algorithms. A review including 28 studies concluded that, in general, non-linear ML models demonstrate a higher precision when compared to the conventional linear models.^62^ However, in cases where traditional methods had already achieved a high accuracy, ML provided no additional clinically significant value.^21^ Nevertheless, an advantage of the ML modelling may pertain to the reduction of the complexity and the cost of the measurements which are required for performing the traditional techniques. Tavallali *et al*.^25^ proposed an ML-based method to estimate cfPWV non-invasively using a single uncalibrated carotid waveform acquired by tonometry in conjunction with a set of routine clinical variables such as age and BP. Their model estimated cfPWV with an RMSE of 1.12 m/s, compared to the reference method.^18^ In addition, authors further supported their findings by showing that estimated PWV was significantly associated with increased risk of future CVD events by using the Framingham database, and this predictive ability was similar to the one by true cfPWV values. Such an approach, along with the high accuracy, offers a less expensive and more convenient way to assess PWV as it does not require the additional measurements of the ECG signal and the femoral pressure tonometry recording which are used in the traditional cfPWV measurement.

### Risk classification

The performance of selected ML-based techniques for vascular risk classification is summarized in *Table 4*. Each study reported the sensitivity and specificity of techniques for classifying patients into two categories, such as whether or not they would experience a CV event. The AUROC statistic, also reported in several studies, combines the sensitivity and specificity to provide a single summary statistic. It varies within the range of 0.0–1.0, where *c*-values of 0.7–0.8 show acceptable discrimination, and values larger than 0.9 show exceptional discrimination. While useful, it should be noted that this statistic can be misleading when the prevalence of the disease is low, such as a low CV event rate, and other statistics such as the positive predictive value provide complementary insights.^64^

**Table 4:** List of selected validation studies of machine learning techniques compared to reference methods for vascular risk classification

Publication	Outcome	Method to assess the outcome	ML technique	Sample size	Age (years)	Sensitivity/ Specificity	AUROC	Externally validated (yes/no)
Alaa *et al.* (2019)^34^	CV event	Blood tests, risk factors	Ensemble of ML pipelines	423 604	56 ± 8	69.9%/—	0.77	No
Al’Aref *et al.* (2020)^28^	Coronary artery disease	Coronary computed tomography angiography, risk factors	Decision tree	13 054	58 ± 11	78%/62.8% and 80%/81.5%	0.77 and 0.88	No
Alty *et al.* (2003)^32^	PWV classification	Photoplethysmogram pulse wave sensor	Support vector machine	5573	—	93%/78%	—	No
Garcia-Carretero *et al.* (2019)^26^	CV event	Tonometry-based PWV, risk factors, laboratory data	Decision tree	88	54 ± 16	98%/95%	—	No
Jamthikar *et al.* (2019)^63^	CV event	Carotid ultrasound, risk factors	Decision tree	202	69 ± 11	9.5%/96.5% and 5.5%/99%	0.80 and 0.68	No
Kakadiaris *et al.* (2018)^30^	CV event	Risk factors	Support vector machine	6459	45–84	86%/95%	0.92	Yes
Sorelli *et al.* (2018)^31^	PW classification	Laser Doppler flowmetry	Support vector machine	54	0–90	65%/90%	0.95	No
Vallée *et al.* (2019)^33^	Coronary heart disease	Tonometry-based PWV, risk factors	Neural network	437	60 ± 11	80%/92%^a^	—	No
Vallée *et al.* (2019)^29^	Coronary heart disease	Tonometry-based PWV, risk factors	Decision tree	530	62 ± 11	82%/85%^a^	0.89	No

AUC, area under the curve; AUROC, area under the receiver operator curve; CV, cardiovascular; ML, machine learning; PW, pulse wave; PWV, pulse wave velocity.

a In the case that more than two classifiers are tested, we report only the results of the best performing classifier.

A key interest in medical research is whether an additional biomarker adds to an existing model. Cook^65^ proposed a reclassification table which indicates the number of subjects who moved to another risk group and the number of those who remained in the same risk group as a result of adding a new predictor. The reclassification concept was extended with the introduction of two metrics, namely, the net reclassification improvement (NRI) and the integrated discrimination improvement (IDI).^66^ An NRI equal to 10% means that subjects with outcome were ∼10% more likely to have an improved reclassification in comparison with subjects with no outcome. An IDI equal to 10% means that the difference in average predicted risks between the subjects with and without the outcome was increased by 10% in the new model. These metrics have been very useful in studies where the performance for different combinations of predictors was assessed or/and the performance of traditional techniques was compared to the performance of novel ML-based methods.^13^^,^^30^ However, prospective studies using reclassification measures to assess the predictive ability of ML-based vascular ageing biomarkers are currently lacking.

Moreover, studies have investigated the potential additive value of ML by comparing the performance of traditional methods to ML-based approaches. A recent study of Desai *et al*. compared several ML models to conventional logistic regression in predicting key heart failure (HF) outcomes.^43^  It was demonstrated that ML improved only slightly the predictive precision. Nevertheless, incorporation of additional parameters from electronic medical records (e.g. laboratory test results as continuous variables) to the ML models showed a competitive advantage over the traditional statistical approach. The authors attributed the much improved performance to the non-parametric nature of the tree-based ML models at making predictions while utilizing continuous variables as inputs. Hence, ML-based approaches might not outperform the conventional modelling in any case, but concurrent refinement of the model’s configuration and feature selection may lead to a superior performance for discriminating several clinical outcomes.

Weng *et al*.,^67^ however, reported improved performance with ML models in comparison to the traditional AHA/ACC risk prediction tool. All ML models had a better predictive capacity at discriminating individuals with or without CV events. An artificial neural network outperformed all the ML models achieving an AUC equal to 0.76.^67^ Ambale-Venkatesh *et al*.^68^ used the longitudinal Multi-Ethnic Study for Atherosclerosis (MESA) cohort study to compare the accuracy between ML-based approaches and the traditional CV risk assessment models (i.e. standard Cox, LASSO-Cox, and AIC-Cox). A large ensemble of 735 variables from imaging, non-invasive tests, questionnaires, and biomarker panels were used as inputs. The outcomes included death, stroke, cardiovascular events, incidents of atrial fibrillation, and HF events.^68^ Authors reported an increase in the *C*-statistic for all outcomes, when they compared their results to the well-established conventional risk scores, including the Framingham and the American College of Cardiology/American Heart Association Atherosclerotic Cardiovascular Disease (ACC/AHA ASCVD) risk scores. In another study, Kakadiaris *et al*.^30^ also used the MESA cohort and demonstrated that their ML Risk Calculator (sensitivity = 0.96, specificity = 0.87, accuracy = 0.89) outperformed that ACC/AHA Risk Calculator (sensitivity = 0.75, specificity = 0.59, accuracy = 0.62) for predicting all CVD events while recommending less drug therapy and missing fewer events.

**Figure 4:**
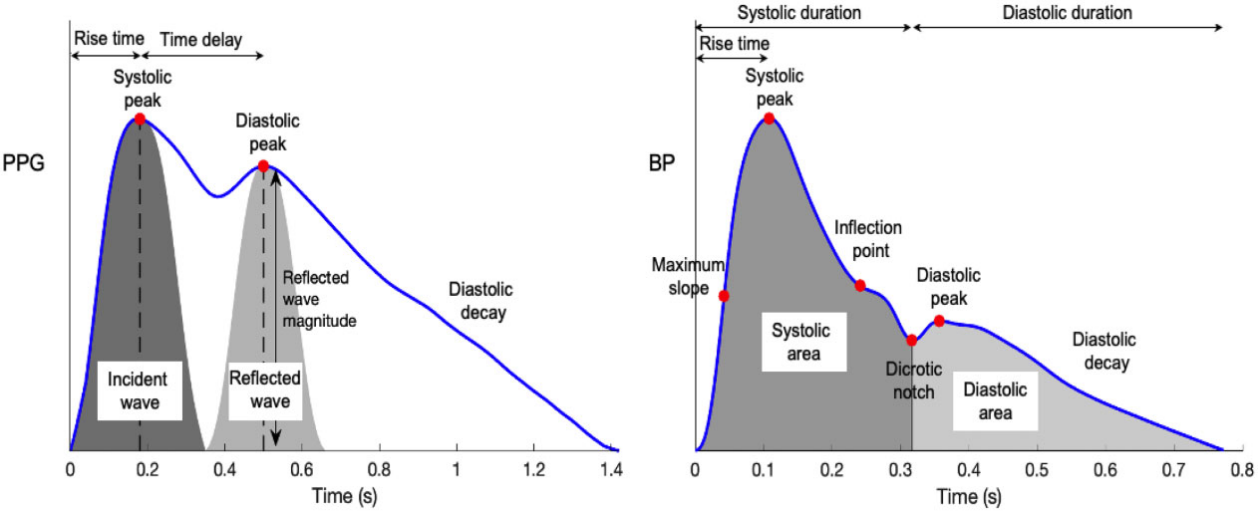
Pulse wave analysis of exemplary photoplethysmography and radial blood pressure waveforms. Adapted from: ‘Photoplethysmogram pulse wave composition’, under CC BY 4.0. BP, blood pressure; PPG, photoplethysmography.

ML models are versatile and can be more flexible compared to traditional risk calculators.^27^^,^^30^ They can combine a plethora of different data sources and lead to more precise and relevant CV risk stratification.^27^ Photoplethysmogram and radial BP pulse waves are commonly used as inputs in ML regression models, as reported in *Table 3*. Moreover, important pulse wave features are presented in *Figure  4*. Classification models use a wider range of inputs, such as images, tonometric signals, and laboratory data (*Table 4*). Finally, ML models can be trained using artificially generated datasets via data augmentation techniques and thus further increase their predictive capacity over the conventional risk assessment techniques.

## The clinical utility of machine learning-based methods

Currently, there is no single correct diagnosis approach for any given patients for CVD prediction due to different clinical characteristics and variability in symptoms of patients and imperfections in results obtained from non-invasive and cardiac tests. Therefore, individual CV risk determination is an important path to take towards a predictive medicine. There is a growing need to find further appropriate, easy to apply, non-invasive tests, and biomarkers that will increase the yield of CVD prediction. However, algorithm conception for correct classification of CVD risk factors remains a major problem.

From a clinical perspective, the data-driven approach of ML may also help optimize pulse wave analaysis algorithms by comparing predictions with data simultaneously obtained through reference standards (typically intra-arterial measurements) and improve the quality assessment of the pulsatile signals. Application of deep learning analysis to ‘big data’ collected through registries may help improve the patient risk stratification and allow accurate long-term risk prediction.

In the contemporary published data, development of ML models and their validation has been demonstrated in a few clinical studies. Initially, the early data were derived from cross-sectional data that provided a proof of concept for researchers to put their algorithms to test with real clinical data (see *Tables 1 and 2*). The ‘one-million dollar’ question is whether the ML-derived estimates of vascular ageing were accurate in estimating the certain vascular ageing biomarker and, of course, whether these ML-derived estimates were at least as prognostic of hard endpoints as their reference method. Although no real conclusions can be made based on the scarce available data on most of the vascular ageing indices, the initial results are promising. In a very elegantly performed study, ambulatory BP measurements and clinical profile were used by Antza *et al.*^69^ to derive an early vascular aging (EVA) ambulatory score comprising 24-h SBP, 24-h DBP, 24-h HR, age, sex, BMI, diabetes mellitus (yes–no), and estimated glomerular filtration rate (modification of diet in renal disease). This score was shown to identify with good accuracy hypertensive patients with EVA that was defined as cfPWV values higher than the expected for age average values according to European population data and further confirmed that the use of scores to identify early vascular ageing is feasible.^70^

In an effort to improve diagnostic accuracy, Vallée *et al*.^33^ used an algorithm based on aortic PWV and ML to better predict CAD. They developed an aPWV index as a measure of an individual patient’s aortic stiffness independent of age, gender, mean BP, and HR. The aPWV index was thus calculated as (measured aPWV − theoretical aPWV)/theoretical aPWV and showed to predict CAD. Furthermore, confirming this strategy of ML-derived indices of vascular ageing were two prospective studies and also data from larger cohorts that assessed coronary calcification score.^28^ The first showed that PWV derived by ML and an uncalibrated trace of carotid pressure waveform is a good prognostic factor of events in the Framingham study.^25^ The second estimated PWV by the Reference Values Equations and showed that ePWV is both capable in predicting events but in sequential measurements could also be used as to monitor treatment efficacy and improve prognosis beyond BP in hypertensives.^13^^,^^14^ However, the events that were mainly predicted by estimated PWV were HF, cerebrovascular events, and all-cause mortality. This confirms the closer link of estimated PWV to events related to arteriosclerosis rather than atherosclerosis.^71^ Therefore, there is a need to identify the appropriate population that will benefit most from the use of ML-based methods such as hypertensives or HF patients,^72^ as well as the most suitable outcomes such as HF and all-cause mortality, as was clearly demonstrated by the recent ambiguous results of the SPARTE trial.^73^

Although ML applications are projected to greatly influence clinical practice, there remains little by way of robust clinical validation of such technologies, and, hence, very few are currently in clinical use. The greatest leap forward in the adoption of ML technologies in clinical practice will be made by ‘translating technical success to meaningful clinical impact’.^74^ This will be aided by establishing methodological frameworks for evaluating and comparing ML tools. Much progress has been made already on this with the TRIPOD statement (see section Reporting machine learning models).

In the near future, it is not science fiction to envisage ML working in the background of standard primary prevention assessment in an outpatient clinic or even through specific applications in a mobile phone or laptop/notebook, gathering the variables automatically and allowing an immediate risk score computation. These methods are already used in everyday practice by many applications that utilize ML secretly that the user is not aware of. An everyday characteristic example is that of web browser advertisements which are based on the passive (unknown to user) collection of parameters and their seamless input into ML algorithms. With the latest advancements in automated feature ranking, ML can be independent of user input and practically fully automated. This is the big step needed to provide a more personalized medicine that will fit each patient’s needs and also support physicians in their everyday practise with on-the-fly answers and solutions specific to the patient. This principle will amalgamate personal characteristics, input from medical equipment/software, and minimal input from physicians to shape the algorithm for each patient.

## Future research directions

### Harnessing electronic health record data

Electronic health records (EHRs) contain a plethora of patient data, ranging from demographic details and clinical notes to laboratory test results and medical images. While EHRs were initially designed to improve the efficiency and accessibility of healthcare systems, they have found varied applications in clinical research,^75^^,^^76^ including cardiovascular event prediction.^77^^,^^78^ In the future EHR data could first be used to identify patients with known risk factors who may benefit from vascular age assessment. Machine learning-based techniques for this purpose would need moderate accuracy to justify the additional clinical workload of assessments. Second, EHR data could be used to estimate vascular ageing parameters which could be used to inform clinical decision-making. Machine learning-based techniques would need a high level of accuracy in this scenario to ensure patient safety.

However, there are limitations to the use of EHR data, including data heterogeneity and model interpretability. For instance, Lauritsen *et al.*^79^ employed various ML models using EHR data for early detection of sepsis, including gradient boosting, multilayer perceptron, and long-term recurrent convolutional networks. While the prediction models performed moderately well, the generalizability of the ML models may be limited. This is likely due to their high dimensional feature space.

### The pulse wave: a gold mine of physiological information

The arterial pulse wave is a rich source of information for assessing vascular health in humans as it is influenced by the cardiac and vascular properties^80^ and thus can reflect physiological changes in the cardiovasculature.^80–82^ Arterial pulse signals are measured in both clinical practice and wearable devices. Two commonly obtained pulse signals are the PPG and radial BP. Numerous physiological parameters can be computed from these signals, which can be useful for health monitoring and clinical decision-making. Previous studies have used an abundance of features extracted from either the PPG or BP waveform (*Figure  4*) and incorporated them into a regression pipeline for the estimation of major vascular biomarkers.^21^^,^^83^^,^^84^ Moreover, further opportunities can arise as deep learning algorithms are capable of revealing more sophisticated pieces of vascular information through learning by themselves from the morphology of the raw physiological signals^85^^,^^86^ without the need for manually extracted features.

### Using consumer devices to assess vascular age in daily life

Research is ongoing to incorporate measures of vascular age into consumer devices such as bathroom scales, smartphones, and wrist-worn fitness trackers.^87–89^ The bathroom scales approach assesses PWV from the time delay between cardiac ejection and arrival of the pulse at the foot, whereas technology for smartphones and fitness trackers assesses vascular age from the shape of a single PPG pulse wave. The use of consumer devices to assess arterial stiffness presents several opportunities: these devices can be used away from the clinical setting, avoiding potential inaccuracies due to white-coat hypertension,^90^ and may facilitate assessment in a range of additional situations, e.g. after exercise,^91^ while asleep, and during potentially stressful daily activities. Results can be fed back to the user immediately and could be used to prompt lifestyle changes. Furthermore, consumer devices can be used remotely, an important consideration in the light of COVID-19. Alternatively, in a clinical setting nurses could be engaged to measure vascular age using novel devices. These methods could provide a relatively easy, cheap, and scalable method for identifying individuals who may benefit from more detailed cardiovascular risk assessment.

However, several challenges remain before the full potential of consumer devices for assessing vascular age can be realized. First, measurements should be contextualized according to the user’s activity: for example, an elevated vascular age measured shortly after exercise would be interpreted differently to a similar assessment during sleep. Algorithms are being developed to detect when a user is sleeping from wearable signals, which could be used to contextualize vascular ageing assessments.^92^ Second, measurements may not be solely indicative of large artery stiffness due to extended PWV path lengths, such as heart-foot PWVs provided by bathroom scales, and the source of pulse wave measurements, such as PPG-derived pulse waves being influenced by the microvasculature. Third, measurements acquired from consumer devices in daily life are more likely to be of low-quality due to motion artefacts and poor sensor contact. Consequently, algorithms are required to reject low-quality data, and prompt the user to reposition the sensor and retake the recording when necessary. Fourth, algorithms are required to post-process the repeated measurements provided by consumer devices in order to condense the data into a manageable summary statistic for clinical use and minimize false alerts. Machine learning provides an approach with which to design such algorithms. Ideally, the summary statistic should be easily comprehensible, comparable to known reference values, and have a biological interpretation. Finally, device design can impact measurements. For instance, wrist-worn devices can differ in their hardware (such as the wavelength of light used by the pulse wave sensor), software (such as filtering and analysis algorithms), and performance (such as agreement between estimated and reference parameters). Consequently, there is a need for standardization of measurement processes where possible, and harmonization of measured parameters to account for any remaining differences between devices.

### A gold standard for vascular age

A reference vascular age is a necessary prerequisite to using supervised ML to develop new models with which to assess vascular age. There are broadly two approaches to defining vascular age: (i) the age of an individual with the same absolute cardiovascular risk but controlled risk factors^93^ or (ii) the age of an individual with the same cardiovascular state, such as arterial stiffness assessed through PWV, but controlled risk factors. However, there is not yet consensus over which approach should be used to calculate a reference vascular age. A widely accepted approach to calculating vascular age supported by strong evidence for its clinical utility would provide a reference with which to train ML models, and justification for using ML models to assess vascular age in clinical practice. A more elaborate method has been recently proposed with the introduction of EVA and the use of an estimation of vascular age based on PWV and its comparison to the true age of each participant.^94^ This approach has the benefit of incorporating age, BP, and treatment in the identification of patients at high CVD risk. A cut-off based on the PWV reference values for certain age, BP, and treatment might be a superior solution, but this remains to be proven in future studies. Finally, in the future, the combination of multiple risk factors analysed with ML methods could improve the prediction of cardiovascular events.

## Conclusion

Vascular ageing biomarkers have been found to be predictive of CV risk independently of classical risk factors, and yet are not widely used in clinical practice. This review highlights the utility of ML for developing new techniques to assess vascular ageing biomarkers quickly and accurately. When coupled with effective interventions these new techniques could help reduce cardiovascular morbidity and mortality. The plethora of data now routinely collected in healthcare settings and in daily life provides opportunity to identify at-risk individuals, to monitor their CV health in daily life, and to assess therapeutic targets. Much further work is required to develop ML-based biomarkers to the required standard for them to be considered as surrogate endpoints of CV events,^95^ and to identify clinical scenarios in which their use is cost-effective.

## Supplementary material

Supplementary material is available at *European Heart Journal – Digital Health*.

## Funding

This paper is based upon work from COST Action CA18216 ‘Network for Research in Vascular Ageing’, supported by COST (European Cooperation in Science and Technology): www.cost.eu . P.H.C. acknowledges funding from the British Heart Foundation (BHF) through grants (PG/15/104/31913) and (FS/20/20/34626), and also the Wellcome EPSRC Centre for Medical Engineering at King's College London (WT 203148/Z/16/Z). R.E.C. is supported by a National Heart Foundation fellowship (102484). V.B. acknowledges funding from the Swiss National Science Foundation (SNF 205321/197234).

**Conflict of interest:** none declared.

## Data availability

The data underlying this article were extracted from the Pulse Wave Database, which is available in Zenodo, at https://dx.doi.org/10.5281/zenodo.2633174. The code used to analyse the data is available in Zenodo, at https://dx.doi.org/10.5281/zenodo.5074026.

## Supplementary Material

ztab089_supplementary_data
