# Supplementary material for: Leveraging the potential of machine learning for assessing vascular ageing: state-of-the-art and future research
Source: Eur Heart J Digit Health. 2021 Oct 18;2(4):676–90. doi: 10.1093/ehjdh/ztab089 (PMC7612526; doi:10.1093/ehjdh/ztab089)
Supplement: ztab089_supplementary_data [file ztab089_supplementary_data.docx]

**Leveraging the potential of machine learning for assessing vascular ageing: state-of-the-art and future research**

Vasiliki Bikia, Terence Fong, Rachel E. Climie, Rosa-Maria Bruno, Bernhard Hametner, Christopher Mayer, Dimitrios Terentes-Printzios, Peter H. Charlton

# **Supplementary Material**

This Supplementary Material provides two further case studies on using machine learning (ML) to assess vascular age, following on from the first case study presented in the main text. It also provides details of how to access the tutorials accompanying each case study.

## **SM1. Case studies**

**SM1.1 Using multiple linear regression to estimate PWV from age and BP**

Aortic pulse wave velocity (PWV) is an independent predictor of cardiovascular morbidity and mortality^1^, but is not yet routinely measured in clinical practice. It has been proposed that aortic PWV could be estimated from two routinely recorded parameters: age and mean blood pressure (MBP)^2^. This may help facilitate the use of PWV for cardiovascular risk assessment in routine practice. In this case study we demonstrate the use of multiple linear regression to derive an equation relating PWV to age and MBP, in a similar manner to the original work^1^.

The data used for this case study were simulated haemodynamic data for 3,837 healthy adult subjects aged from 25 to 75 years old (in 10-year intervals) from the Pulse Wave Database (PWDB)^3^. The subjects all had different cardiovascular properties within normal ranges (including arterial stiffness, blood pressure, aortic diameter, and stroke volume). The dataset exhibited increases in PWV with age and blood pressure (shown in *Figure SM1*, top panel). Linear regression was used to establish a formula relating the inputs (age and BP) to the output (PWV). A multiple linear regression model was trained using 50% of the dataset to estimate PWV from age and MBP. The model incorporated an intercept, linear terms (age and MBP), quadratic terms (age^2^ and MBP^2^), and a linear interaction term (age x MBP). The resulting equation relating PWV to age and MBP was:

PWV = 1.38 - 0.0747 x age + 0.0724 x MBP + 0.000773 x age x MBP + 0.0008 x age^2^ - 0.000235 x MBP^2^

The lower panel of *Figure SM1* shows a comparison between the reference and estimated PWVs on the remaining 50% of the dataset. The limits of agreement between the estimated and reference PWVs (within which 95% of errors would be expected to lie) were 0.0 ± 2.2 m/s. The largest errors were observed in subjects aged 55 and older with elevated PWVs, for whom the model tended to underestimate PWV.


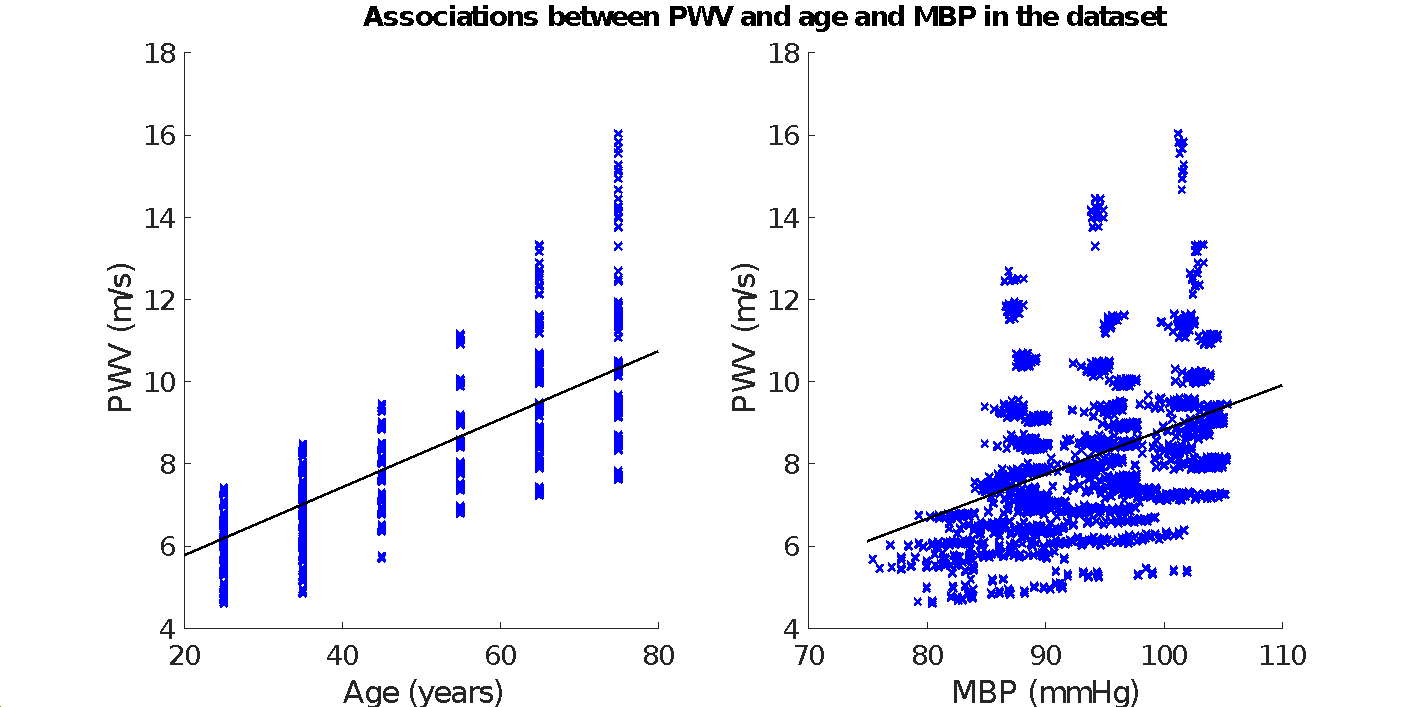


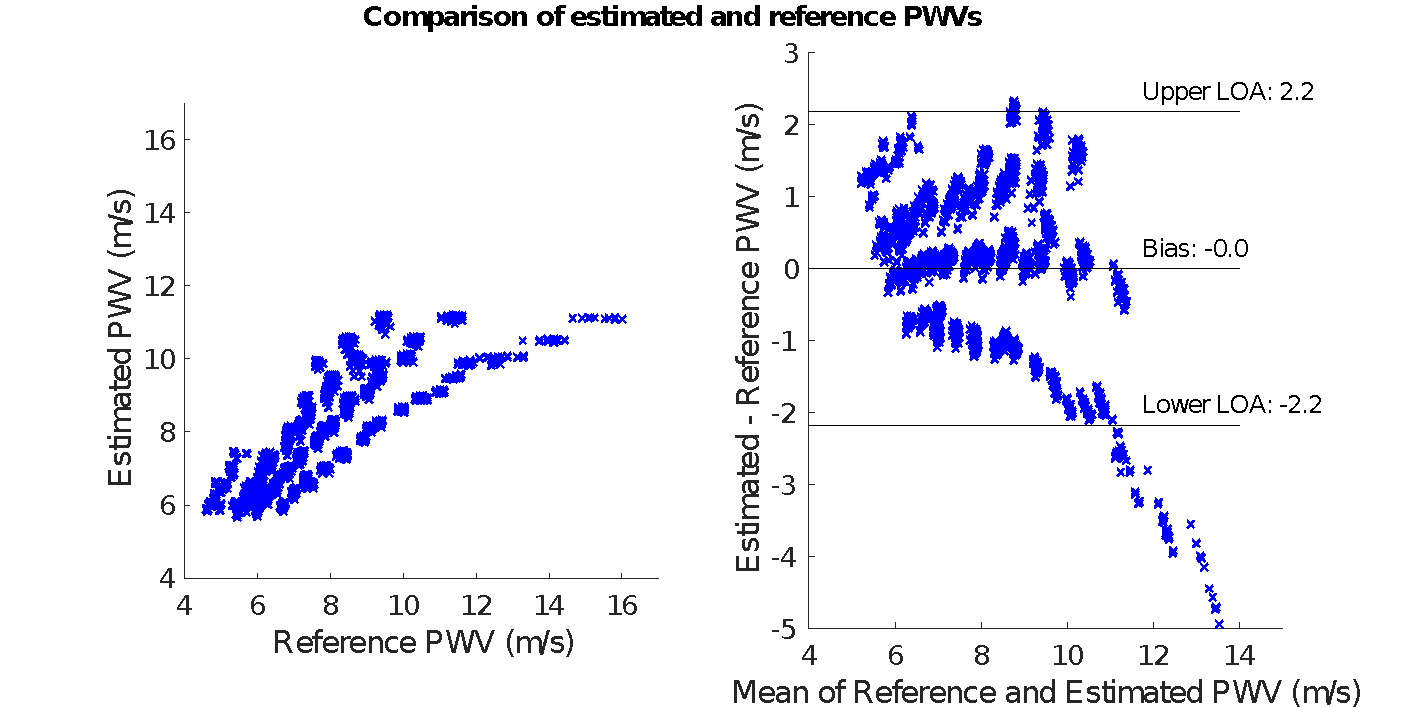


*Figure SM1:* A case study of estimating pulse wave velocity (PWV) from age and mean blood pressure (MBP) using multiple linear regression.

This case study demonstrates one application of ML to estimate a target parameter from more easily measured parameters. In this instance, carotid-femoral PWV is not routinely acquired, but it has been proposed that a model developed using ML can provide estimates of this risk factor which could enhance cardiovascular risk assessment^2^. The assessment of performance on the training data indicates that errors can be considerable even with ideal data, highlighting a limitation of ML.

**SM1.2 Using a neural network to assess vascular age from pulse waves**

The widespread use of wrist-worn wearables provides a new opportunity for cardiovascular monitoring in daily life. Many wearables measure the arterial pulse wave for heart rate monitoring using an optical photoplethysmogram (PPG) sensor. The PPG could potentially also be used to assess vascular age, as the PPG pulse wave shape changes with age^4^ as shown in *Figure SM2(A)*. In this case study we demonstrate the use of a deep learning neural network to classify PPG pulse waves as either young or elderly.

**Healthy volunteer data Simulated data**

(a)
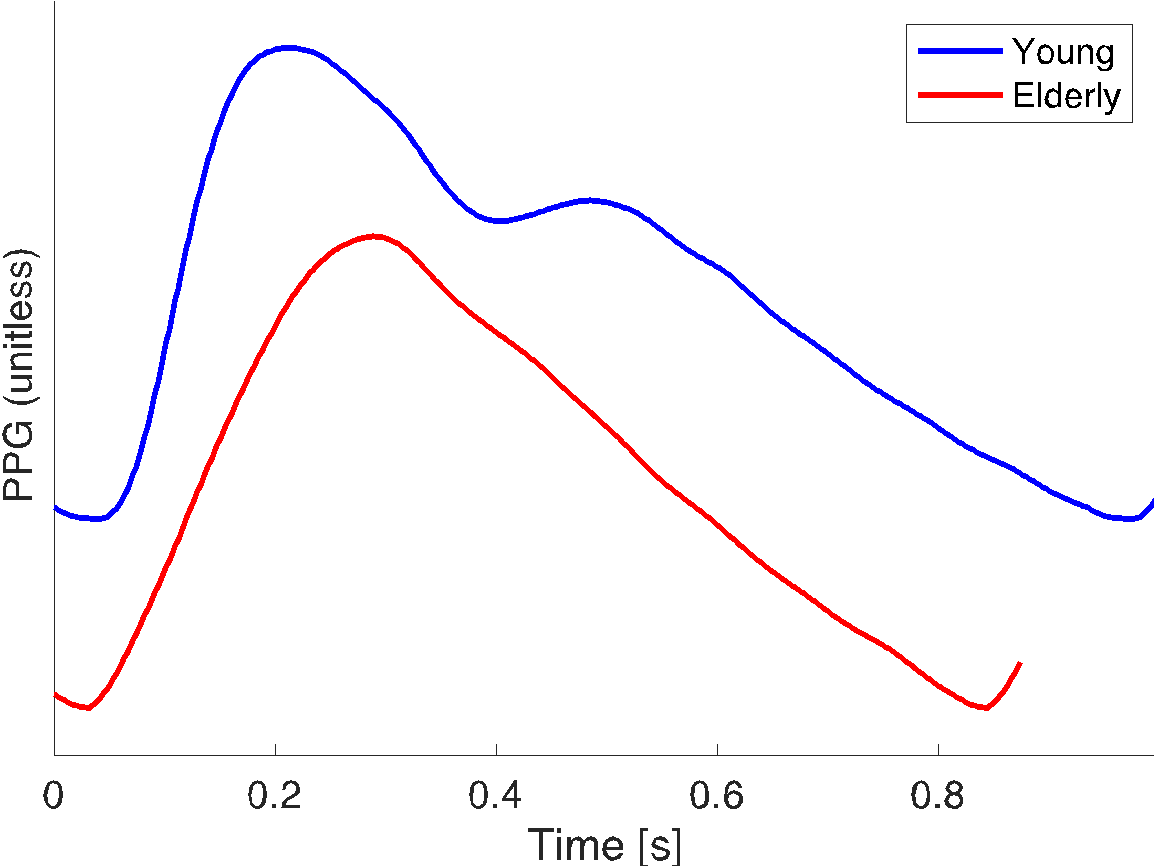
 (b)
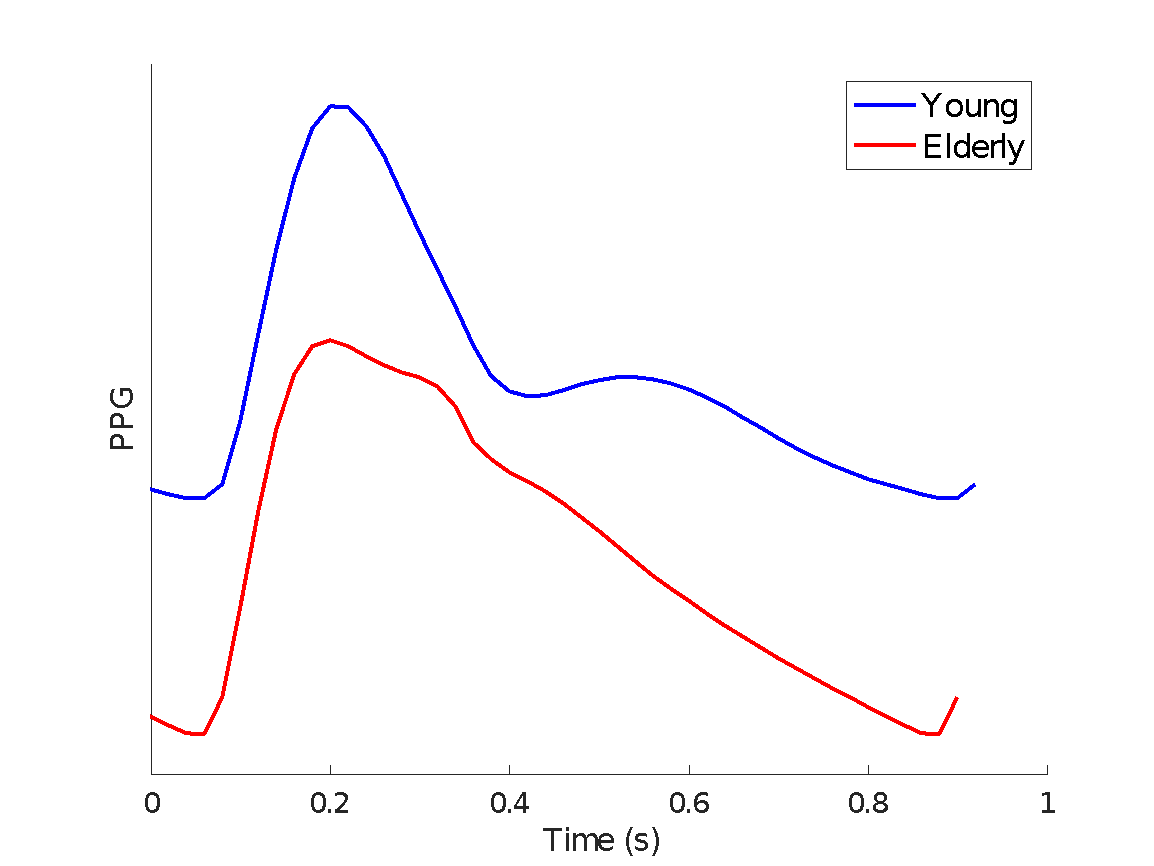


*Figure SM2:* A comparison of photoplethysmogram (PPG) pulse wave shapes between young and elderly subjects: (A) data from healthy volunteers (from the Vortal Dataset^5^); (B) examples of simulated PPG pulse wave data used in the case study.

The data used in this case study were simulated PPG pulse waves for young and elderly subjects (aged 25 and 75 years old) with a range of cardiovascular properties. The data were extracted from the PWDB database - the database used in the first case study^3^. The simulated PPG pulse waves exhibited similar changes in shape with age to that observed *in vivo*, as shown by the comparison in *Figure SM2* between (A) healthy volunteer data and (B) simulated data. The data were split into training and testing subsets: each subset contained data from 635 subjects: 356 young subjects and 279 elderly subjects. A neural network with 100 hidden layers was trained to classify PPG pulse waves as either young or elderly.

The neural network classified 608 out of the 635 testing subset pulse waves correctly: an accuracy of 95.7%. The 27 incorrectly classified pulse waves were all elderly subjects who were misclassified as young subjects: interestingly, these all had low cardiac outputs. This highlights a challenge in estimating vascular age from the PPG: both vascular and cardiac properties can affect the PPG pulse wave. Therefore, care should be taken to ensure methods to assess vascular age from the PPG remain accurate across a range of cardiac properties.

## **SM2. Tutorials**

The case studies are accompanied by tutorials on using ML to assess vascular age. The tutorials allow the novice to reproduce the results of the case studies. Each tutorial is provided as a Matlab script, containing step-by-step instructions guiding the user through the analysis. Links are provided to the required datasets, which are publicly available. The latest versions of the tutorials can be found at the following GitHub repository: <https://github.com/peterhcharlton/VascAgeTutorials>

### **SM2.1. Using a random forest regressor to estimate central BP from age and cuff BP**

This case study (described in the main text) can be reproduced using:

- The ‘estimating_age_cbp_bbp.mlx’ Matlab script available at: <https://doi.org/10.5281/zenodo.5074026>
- The ‘pwdb_data.mat’ Pulse Wave Database file available at: <https://doi.org/10.5281/zenodo.3275625> .

### **SM2.2. Using multiple linear regression to estimate PWV from age and BP**

This case study can be reproduced using:

- The ‘estimating_pwv_age_bp.mlx’ Matlab script available at: <https://doi.org/10.5281/zenodo.5074026>
- The ‘pwdb_data.mat’ Pulse Wave Database file available at: <https://doi.org/10.5281/zenodo.3275625> .

### **SM2.3. Using a neural network to assess vascular age from pulse waves**

This case study can be reproduced using:

- The ‘classifying_ppg_age.mlx’ Matlab script available at: <https://doi.org/10.5281/zenodo.5074026>
- The ‘pwdb_data.mat’ Pulse Wave Database file available at: <https://doi.org/10.5281/zenodo.3275625> .

# **Supplementary References**

1. Vlachopoulos C, Aznaouridis K, Stefanadis C. Prediction of cardiovascular events and all-cause mortality with arterial stiffness: a systematic review and meta-analysis. Journal of the American College of Cardiology 2010;**55**(13):1318-1327.

2. Greve SV, Blicher MK, Kruger R, Sehestedt T, Gram-Kampmann E, Rasmussen S, Vishram JKK, Boutouyrie P, Laurent S, Olsen MH. Estimated carotid–femoral pulse wave velocity has similar predictive value as measured carotid–femoral pulse wave velocity. Journal of Hypertension 2016;**34**(7):1279-1289.

3. Charlton PH, Harana JM, Vennin S, Li Y, Chowienczyk P, Alastruey J. Modeling arterial pulse waves in healthy aging: a database for in silico evaluation of hemodynamics and pulse wave indexes. American Journal of Physiology-Heart and Circulatory Physiology 2019;**317**(5):H1062-H1085.

4. Allen J, Murray A. Age-related changes in the characteristics of the photoplethysmographic pulse shape at various body sites. Physiological Measurement 2003;**24**(2):297-307.

5. Charlton PH, Bonnici T; Tarassenko L, Alastruey J, Clifton DA, Beale R, Watkinson PJ. Extraction of respiratory signals from the electrocardiogram and photoplethysmogram: technical and physiological determinants. Physiological Measurement 2017; **38**(5):669–690.
